# A bibliometric analysis of strabismus (from 2004 to 2023)

**DOI:** 10.3389/fmed.2025.1488817

**Published:** 2025-04-28

**Authors:** Yanghui Xiu, Yujie Zhang, Yu Su, Chufan Zhu, Zhen Liu

**Affiliations:** ^1^Affiliated Xiamen Eye Center and Eye Institute, School of Medicine, Xiamen University, Xiamen, Fujian, China; ^2^Xiamen Clinical Research Center for Eye Diseases, Xiamen, Fujian, China; ^3^Xiamen Key Laboratory of Ophthalmology, Xiamen, Fujian, China; ^4^Fujian Key Laboratory of Corneal & Ocular Surface Diseases, Xiamen, Fujian, China; ^5^Xiamen Key Laboratory of Corneal & Ocular Surface Diseases, Xiamen, Fujian, China; ^6^Translational Medicine Institute of Xiamen Eye Center of Xiamen University, Xiamen, Fujian, China; ^7^Longyan Eye Hospital, Xiamen Eye Center and Eye Institute of Xiamen University, Xiamen, China

**Keywords:** strabismus, bibliometrics analysis, Web of Science Core Collection, CiteSpace, research hotspots

## Abstract

Over the past two decades, strabismus research has evolved significantly, driven by innovations in AI-assisted diagnostics, minimally invasive surgical techniques, and a heightened focus on psychosocial outcomes and systemic disease linkages. This bibliometric analysis of 4,515 articles from the Web of Science Core Collection (2004–2023) maps global research trends, identifying the United States of America (USA), China, and the United Kingdom (UK) as leading contributors. A keyword co-occurrence analysis highlights a shift toward innovative treatments, including non-surgical interventions, and highlights the growing interest in interdisciplinary approaches that integrate clinical practice with psychological and social dimensions of strabismus. In conclusion, this bibliometric review provides a comprehensive overview of current strabismus research and identifies key areas for further investigation, serving as a valuable resource for researchers and clinicians aiming to advance the field.

## Introduction

Strabismus, characterized by ocular misalignment, is a clinically significant disorder that impairs binocular vision, depth perception, and psychosocial wellbeing, with profound implications for quality of life (1). Globally, its prevalence ranges from 0.14 to 5.65%, underscoring its status as a major public health concern in ophthalmology (2).

While advancements in surgical techniques (e.g., adjustable sutures and minimally invasive approaches) (3), non-surgical interventions (e.g., prismatic correction and vision therapy) (4–6), and a growing recognition of its psychosocial sequelae (7) have improved clinical outcomes, critical challenges persist. First, there is incomplete elucidation of the pathophysiological mechanisms underlying strabismus, particularly genetic and neurodevelopmental contributors (8). Second, the limited understanding of new surgical techniques for strabismus has led to the emergence of some urgent issues in clinical practice that need to be addressed and resolved (3). Moreover, the growing interdisciplinary nature of strabismus research currently incorporates fields such as psychology, public health, and advanced imaging technologies (9, 10). These gaps necessitate a systematic synthesis of existing knowledge to identify research priorities and guide future investigations.

The exponential growth of scientific literature poses significant challenges for researchers seeking to navigate evolving trends in specialized fields such as strabismus (11). Bibliometrics, a quantitative methodology for analyzing publication patterns, authorship networks, and keyword dynamics, has emerged as a powerful tool to map scientific landscapes (12). By leveraging large-scale data from databases such as the Web of Science or Scopus, bibliometric analyses enable the identification of research hotspots, collaborative clusters, and emerging frontiers while also revealing understudied areas (13). This approach has been successfully applied in ophthalmology to evaluate trends in dry eye (14), uveitis (15), and artificial intelligence in ophthalmology (16) but remains underutilized in strabismus research.

In this study, we conduct the first comprehensive bibliometric analysis of global strabismus research from 2004 to 2023. Using data extracted from the Web of Science Core Collection (WOSCC), we quantified publication trends, mapped international collaboration networks, and performed keyword co-occurrence analysis to identify thematic clusters. Additionally, we used burst detection algorithms to pinpoint transformative studies and temporal shifts in research focus. We delineated the evolution of strabismus research over two decades and forecasted future directions, including the integration of artificial intelligence in diagnostics and novel therapeutic paradigms. This analysis aims to provide clinicians and researchers with an evidence-based roadmap for advancing the field.

## Methods

**Figure 1:**
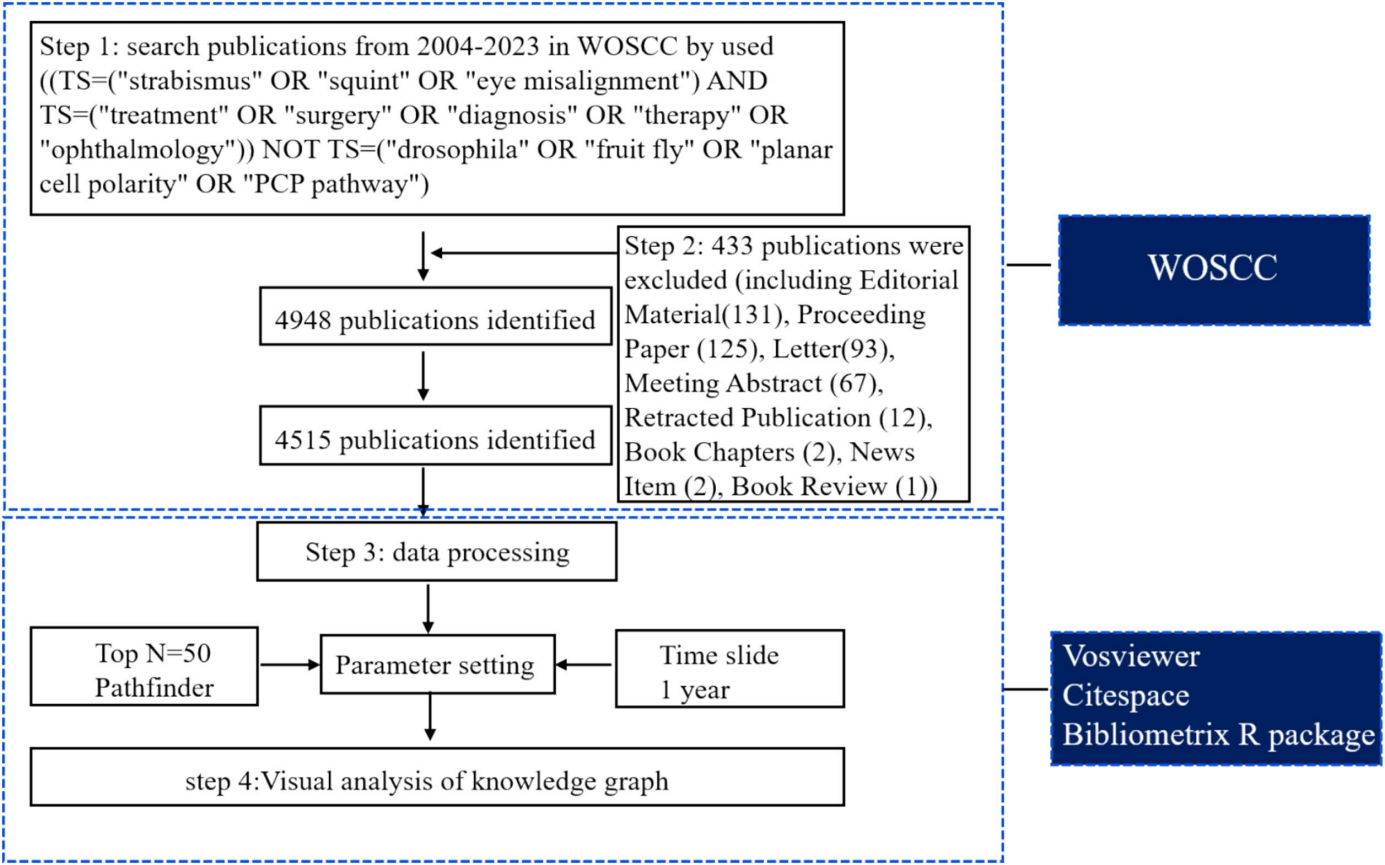
Step-by-step literature process.

The data source was obtained from the WOSCC database, a widely used platform for bibliometric analyses that provides comprehensive bibliometric information. A search was conducted in the WOSCC database on 18 January 2024, covering the past two decades (2004–2023). The initial search yielded a total of 4,948 publications. After excluding editorial materials (131), proceeding papers (125), letters (93), meeting abstracts (67), retracted publications (12), book chapters (2), news items (1), meetings (2), and book reviews (1), a total of 4,515 articles and reviews were included in the bibliometric visualization analysis. The bibliometric information was exported as a “full record and cited references” and analyzed using the Bibliometrix R package (17), VOSviewer (version 1.6.18), and CiteSpace (V.6.2. R3). These software tools are widely used for knowledge mapping and visual analysis to identify current research hotspots and trends. The parameters were determined as follows: top N = 50, clipping = Pathfinder, time slice = 1 year, and clustering algorithm = log-likelihood ratio (LLR). The study framework is outlined in Figure 1.

## Results

### General statistics

**Figure 2:**
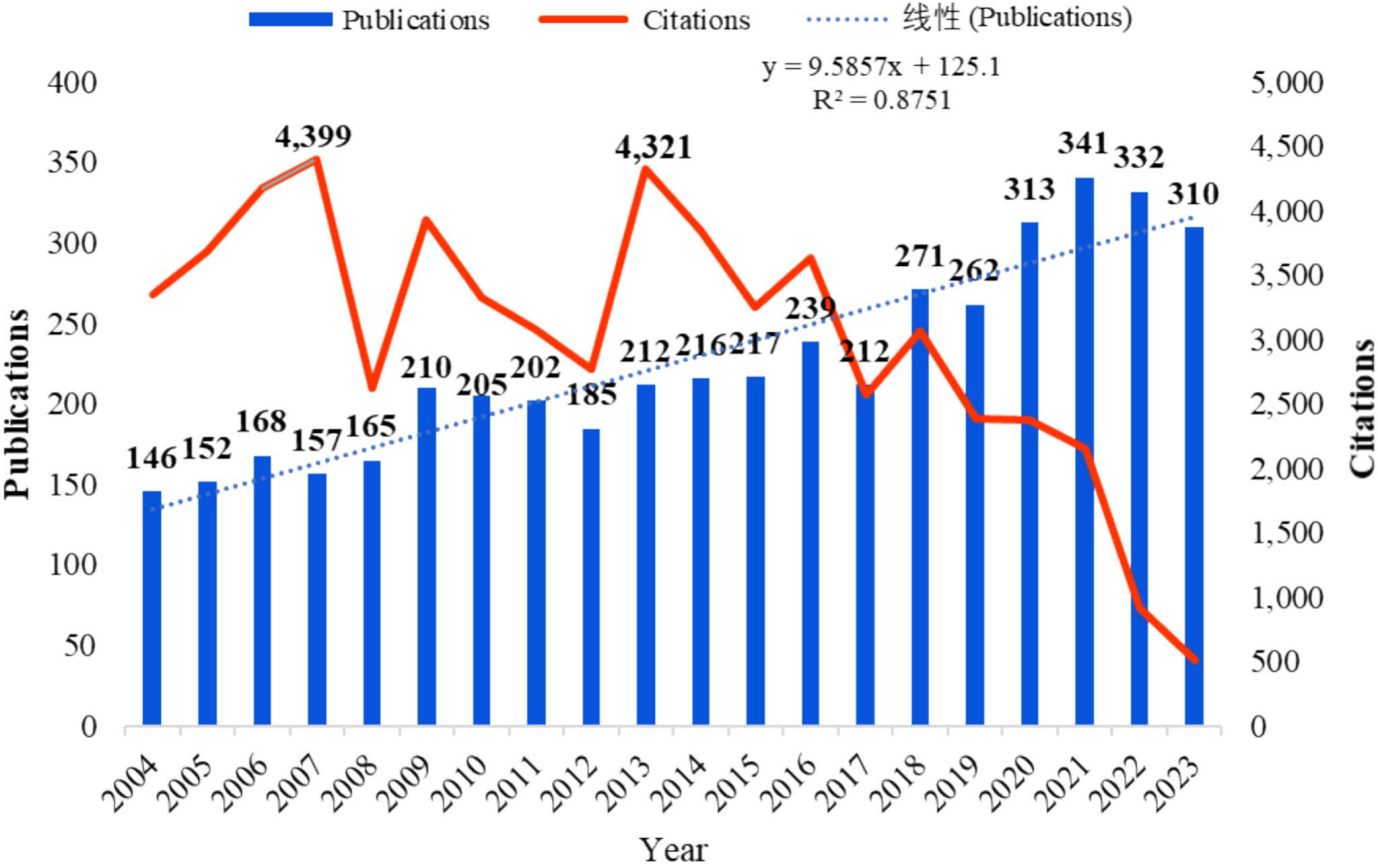
Annual publications and citations over time.

The bibliometric analysis of 4,515 publications on strabismus research (2004–2023) retrieved from the WOSCC revealed multifaceted research dynamics. These publications originated from 146 countries/regions, involved 14,394 authors, and were published across 663 journals (Table 1). A comprehensive analysis of these publications uncovered 5,929 keywords, and the majority of them were written in English. The field exhibited sustained growth, reaching an annual peak of 286 publications in 2021 (Figure 2). On average, each document received 15.31 citations, with the highest citations recorded in 2007 (4,399 citations) and 2013 (4,321 citations). However, a decline in the number of citations has been observed in recent years, possibly due to the limited time available for newer publications to accumulate citations, although noteworthy discoveries may still have been made.

**Table 1:** Main information about the data.

Description	Results
Main information about the data	
Timespan	2004:2023
Sources (journals, books, etc.)	663
Documents	4,515
Annual growth rate %	−5.71
Document average age	10
Average citations per doc	15.31
References	70,443
Document contents	
Keywords Plus (ID)	5,929
Author’s keywords (DE)	5,704
Authors	
Authors	14,394
Authors of single-authored docs	182
Authors collaboration	
Single-authored docs	236
Co-authors per doc	5.02
International co-authorships %	14.11

### Global contribution of countries/regions to the field

**Figure 3:**
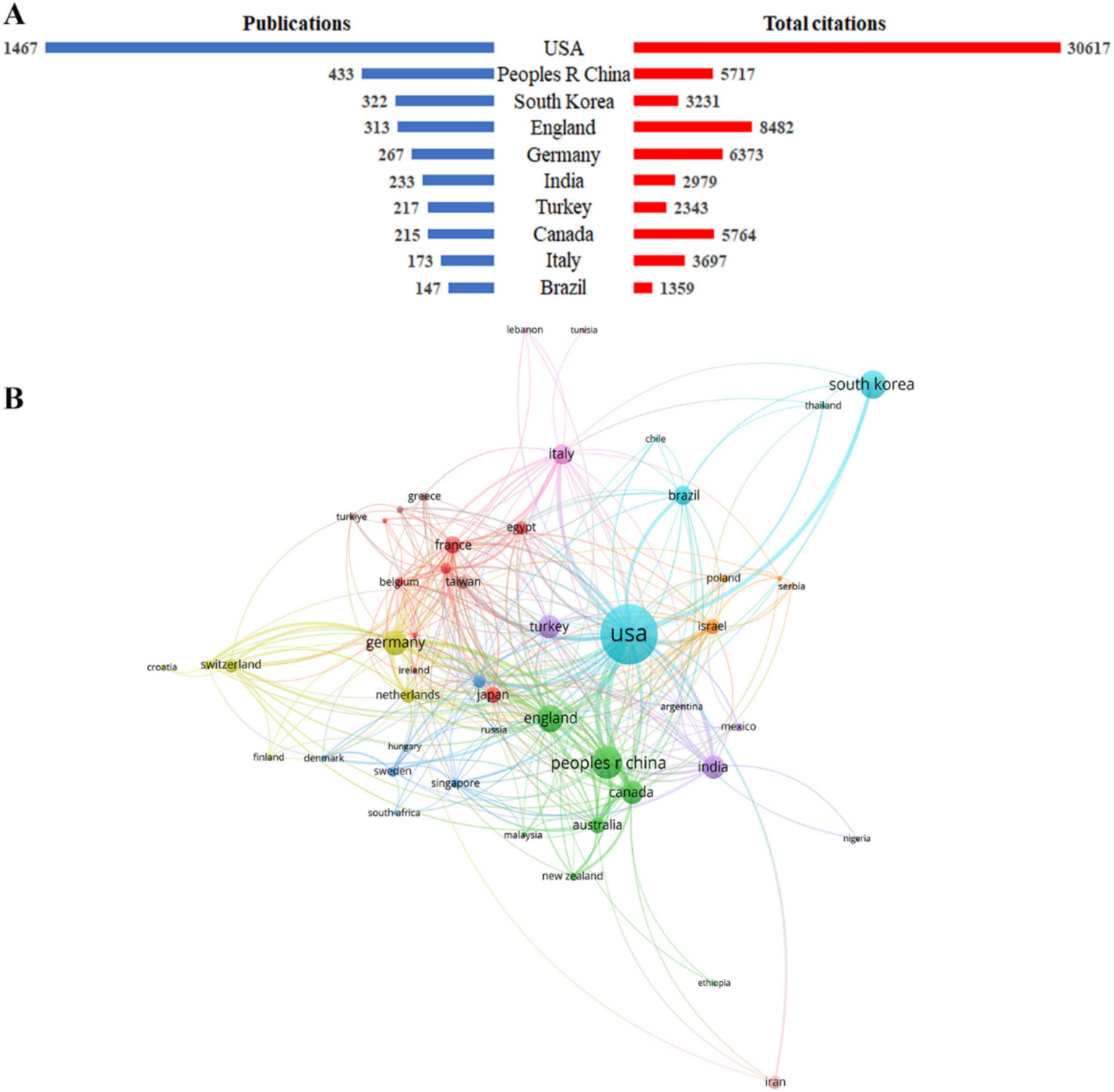
Main countries/regions distribution of strabismus publications. **(A)** The top 10 most productive countries in the publication of strabismus and their corresponding total citations. **(B)** Academic collaboration between different countries/regions.

Between 2004 and 2023, a total of 146 countries/regions published strabismus-related articles. The United States of America (USA) contributed the highest number of publications (1,467), followed by China (433) and South Korea (322). The USA also dominated in total citations (30,617), average citations per article (20.87), and H-index (69), reflecting its substantial scholarly influence. England ranked second in total citations (8,482) and achieved the highest average citations per article (27.10), underscoring its impactful contributions (Figure 3A; Table 2). Collaborative networks, visualized via VOSviewer, revealed robust international partnerships among leading countries (Figure 3B). Node dimensions corresponded to publication volume, connection thickness reflected collaboration intensity, and colors differentiated clusters of closely interacting entities. Notably, all of the top 10 countries by publication count exhibited strong cooperative ties, emphasizing the interconnected nature of global strabismus research.

**Table 2:** Top 10 most productive countries.

Countries	Publications	Centrality	Total citations	Av. article citations	H-index
USA	1,467	0.08	30,617	20.87	69
PEOPLE’S REPUBLIC OF CHINA	433	0.03	5,717	13.2	31
SOUTH KOREA	322	0	3,231	10.03	25
ENGLAND	313	0.07	8,482	27.1	46
GERMANY	267	0	6,373	23.87	30
INDIA	233	0.03	2,979	12.79	27
TURKEY	217	0	2,343	10.8	23
CANADA	215	0.06	5,764	26.81	34
ITALY	173	0.07	3,697	21.37	26
BRAZIL	147	0	1,359	9.24	19

The H-index, a widely recognized metric introduced by Hirsch (18), quantifies both the productivity and citation impact of a researcher or country. Specifically, an H-index of *n* indicates that *n* publications have each been cited at least *n* times. For instance, the USA’s H-index of 69 signifies that 69 of its articles have received ≥69 citations each, highlighting its dual strength in output volume and sustained academic influence. Centrality is a measure of a node’s importance within a co-occurrence or co-citation network. A node with high centrality acts as a critical junction, facilitating the flow of information and collaboration across the network (19). In our study, centrality values were computed to assess the strategic positioning of countries in the global strabismus research network. The USA (centrality = 0.08), England (0.07), and Italy (0.07) emerged as pivotal hubs, indicating their roles in fostering cross-regional collaborations and disseminating knowledge. These metrics collectively underscore the USA’s central role in advancing strabismus research, both in terms of output quality (H-index = 69) and collaborative influence (centrality = 0.08). The integration of productivity, citation impact, and network positioning provides a comprehensive assessment of a nation’s scholarly footprint.

### Institutional contributions and collaborative networks in strabismus research

**Figure 4:**
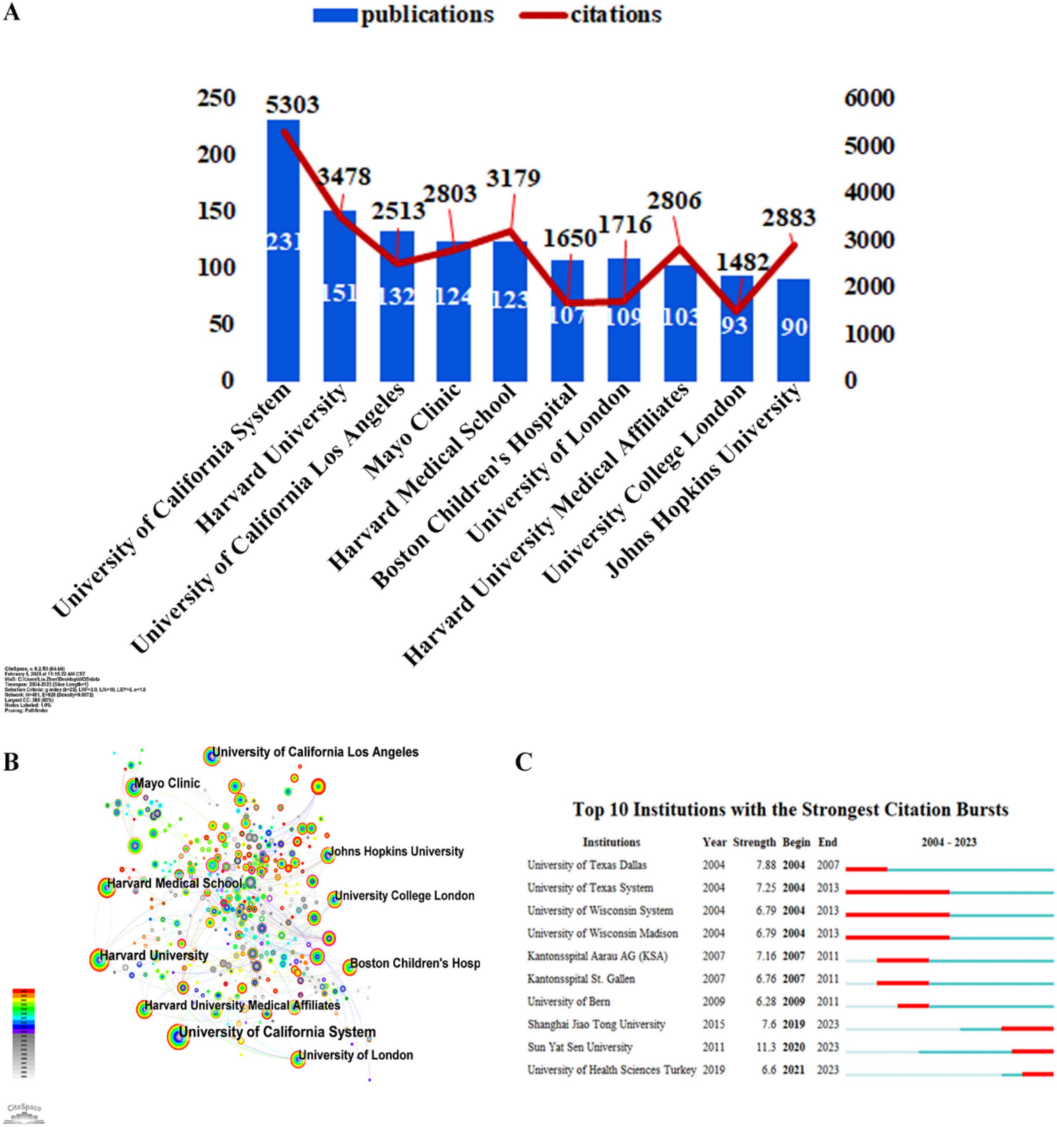
Main institutions distribution for strabismus publications. **(A)** The top 10 most productive institutions in the publication of strabismus and their corresponding total citations. **(B)** Academic collaboration between different institutions. **(C)** The top 10 institutions with the strongest citation bursts. The red bold line represents the burst years.

A total of 481 institutions worldwide have contributed to research in the field of strabismus. Analysis of the leading institutions revealed a clear dominance of U.S.-based entities, with eight of the top 10 institutions originating from the USA and two from England. The University of California (UC) system emerged as the most prolific contributor, producing 231 publications (5,303 citations), followed by Harvard University with 151 publications (3,478 citations). Other leading institutions include the University of California, Los Angeles (UCLA), Mayo Clinic, Harvard Medical School, Boston Children’s Hospital, University of London, Harvard University Medical Affiliates, University College London (UCL), and Johns Hopkins University (Figure 4A).

To map institutional collaborations, a network analysis was performed using CiteSpace (Figure 4B). Nodes represent institutions, with node size proportional to publication output and connecting lines denoting collaborative relationships. Burst detection analysis was used to identify institutions with temporally intensified research activity. Notably, Sun Yat-Sen University exhibited the strongest citation burst from 2020 to 2023, indicating a significant surge in strabismus-related research during this period (Figure 4C). Among the top 10 institutions with citation bursts, two were based in China, reflecting the country’s emerging role in advancing global strabismus research.

### Journals and authors

**Figure 5:**
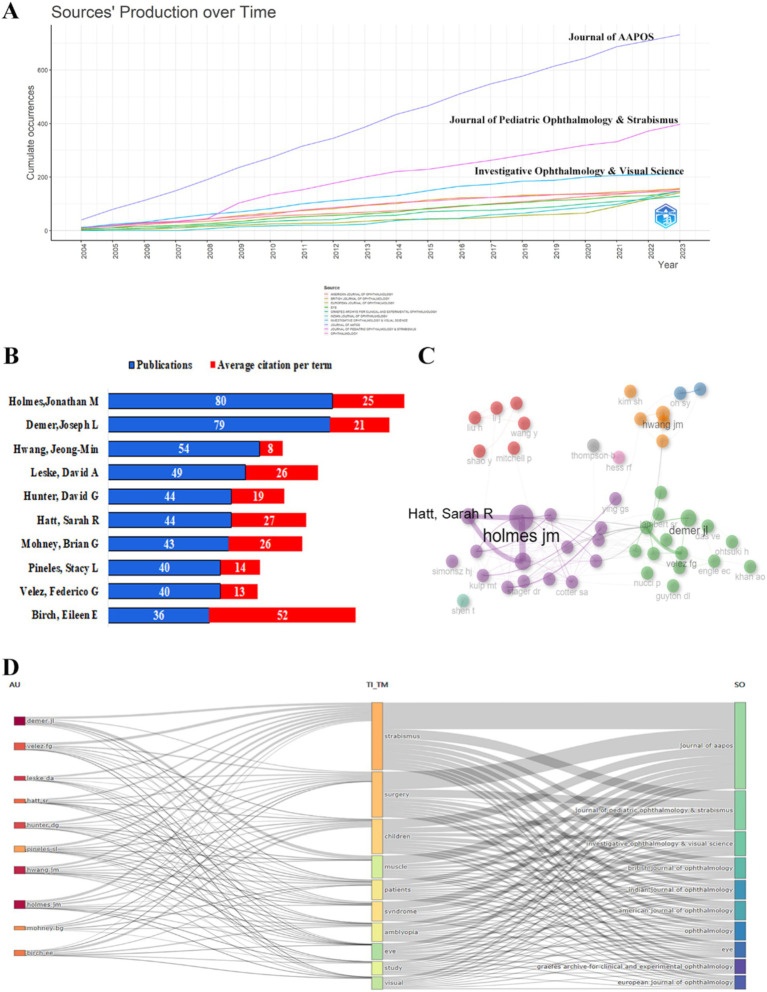
Visualization map of journals and authors in the field of strabismus from 2004 to 2023. **(A)** Top journals’ production over time. **(B)** Publications and average citation per term of the most contributing authors. **(C)** Co-authorship network of the authors. **(D)** A Sankey diagram between authors, topics, and journals.

A total of 4,515 publications were documented across 663 journals, with the top 10 journals contributing 40.91% of overall publications (Table 3). Notably, the majority of these leading journals were based in the USA, with three journals from Europe and the remaining journals originating from India and Germany. The *Journal of the American Association for Pediatric Ophthalmology and Strabismus* (AAPOS) emerged as the leading publisher with 590 articles, followed by *the Journal of Pediatric Ophthalmology & Strabismus* (311) and *Ophthalmology* (136). Notably, the publication frequency did not correlate with academic impact metrics. For example, *Ophthalmology* demonstrated exceptional influence with the highest average citations per article (40.3 citations/item), the highest H-index (44), and the greatest citation density, despite only ranking third in publication volume. Other high-impact journals included I*nvestigative Ophthalmology & Visual Science* (28.17 citations/item) and the *British Journal of Ophthalmology* (22.33 citations/item) (Table 4). The volume of literature in strabismus research has shown a consistent upward trend over the past decade (Figure 5A). Among the leading journal in the field, the Journal of AAPOS played a pivotal role in the discipline’s early development. Notably, its publication output has accelerated significantly over the past decade, further solidifying its influential position.

**Table 3:** Top 10 journals ranked by their publications in the strabismus area.

Rank	Source	Documents	Citations	Average per item	H-index	IF
1	Journal of AAPOS	590	8,214	13.92	42	1.6
2	Journal of Pediatric Ophthalmology & Strabismus	311	2,255	7.25	24	1.2
7	Ophthalmology	136	5,481	40.3	44	13.7
6	Indian Journal of Ophthalmology	129	987	7.65	19	3.1
5	American Journal of Ophthalmology	125	2,100	16.8	28	4.2
9	European Journal of Ophthalmology	123	509	4.13	12	1.7
4	British Journal of Ophthalmology	122	2,725	22.33	30	4.1
8	Eye	119	1,715	14.41	25	3.9
10	Graefe’s Archive for Clinical and Experimental Ophthalmology	103	936	9.08	16	2.4
3	Investigative Ophthalmology & Visual Science	89	2,507	28.17	28	4.4

**Table 4:** Keyword frequency and centrality statistics.

Keyword frequency statistics			Keyword centrality statistics		
Rank	Keywords	Frequency	Rank	Keywords	Centrality
1	children	660	1	diagnosis	0.09
2	strabismus surgery	571	2	strabismus surgery	0.08
3	management	338	3	management	0.07
4	prevalence	283	4	extraocular muscles	0.07
5	amblyopia	202	5	anesthesia	0.07
6	risk factors	161	6	recession	0.06
7	vision	157	7	age	0.06
8	visual acuity	151	8	complications	0.06
9	diagnosis	148	9	therapy	0.06
10	recession	144	10	risk	0.06
11	intermittent exotropia	141	11	infantile esotropia	0.06
12	age	135	12	follow-up	0.06
13	impact	123	13	surgical management	0.06
14	quality of life	111	14	extraocular muscle	0.06
15	adults	101	15	botulinum toxin	0.05

A total of 14,394 authors contributed to strabismus research, highlighting the field’s collaborative nature. Figure 5B shows the top 10 most prolific scholars in strabismus from 2004 to 2023. Jonathan M. Holmes was the leading author, with 69 publications and an average of 29 citations per article, reflecting the significant impact of his study. Joseph L. Demer followed with 54 publications and an average of 22 citations per article, indicating his considerable influence as well. High citation counts often reflect the quality and relevance of research, as they suggest that these studies have been widely referenced and built upon. Co-authorship network analysis identified three distinct research clusters (Figure 5C). The first cluster, led by Holmes and Leske, focused on developing standardized diagnostic frameworks for pediatric and adult strabismus. The second cluster, centered around Velez, specialized in the surgical management of complex strabismus, including adjustable suture techniques. The third cluster, under Hwang, explored epidemiological studies on strabismus in East Asian populations, integrating genetic and intervention outcome analyses. Additionally, an analysis of the top 10 most productive revealed that their work was primarily published in the Journal of AAPOS (Figure 5D).

### Analysis of keyword co-occurrence

Keyword co-occurrence analysis provides valuable insights into emerging trends in strabismus research. High-frequency keywords indicate areas of concentrated interest while centrality metrics highlight their bridging roles in the knowledge network. Among the top 15 keywords (Table 4) “Children” has the highest frequency (excluding “Strabismus”) but low centrality suggesting fragmented research in pediatric populations. In contrast “Diagnosis” (centrality: 0.09) and “Strabismus Surgery” (centrality: 0.08) have strong interdisciplinary connections reflecting their importance in advancing diagnostic and surgical approaches.

**Figure 6:**
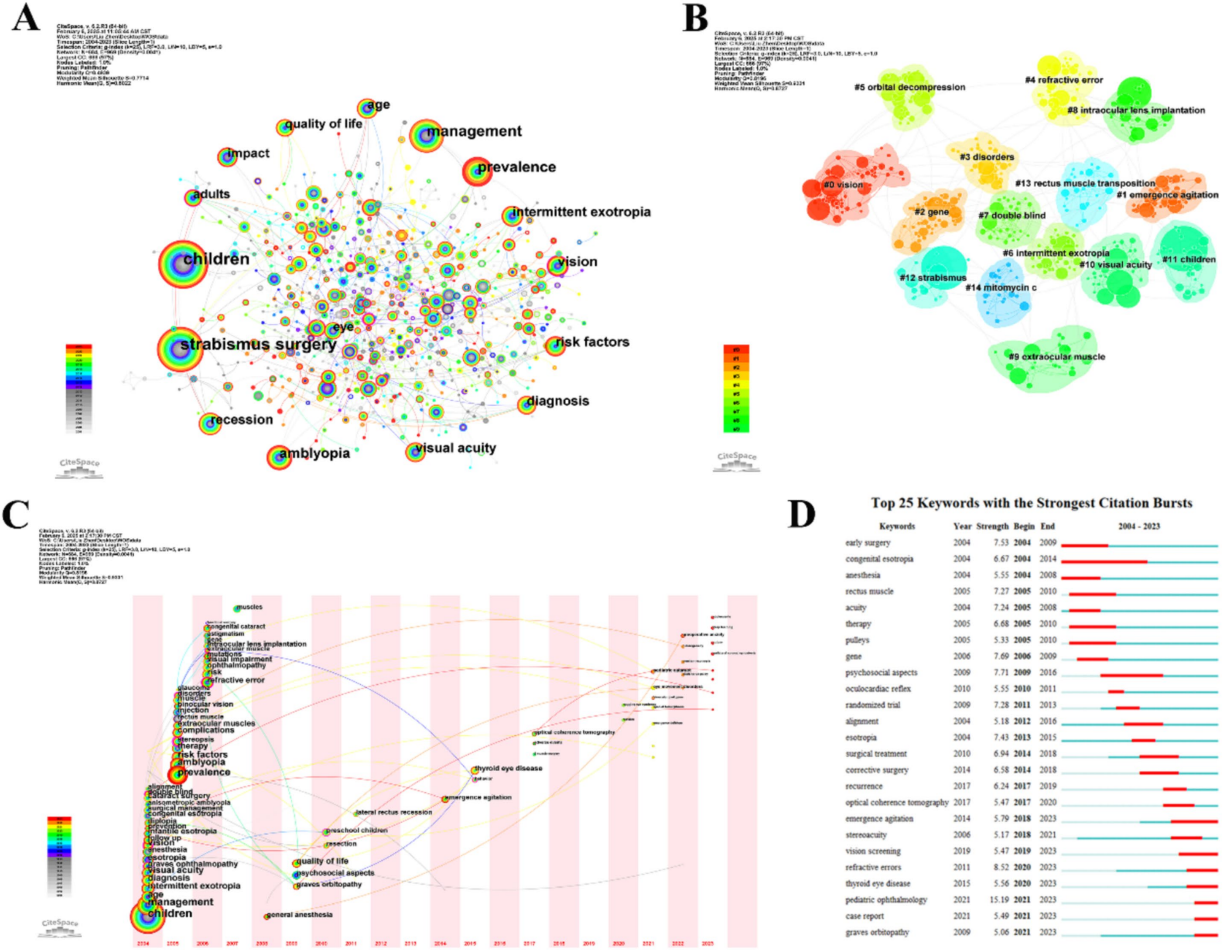
Visualization of keyword analysis in the field of strabismus from 2004 to 2023. **(A)** Visualization network map of keywords. **(B)** Keyword co-occurrence cluster map based on different research domains. **(C)** Keywords time zone map of related literature. The time zone diagram progresses from left to right, with the years increasing. Keywords that first appear in the same year are grouped within the same time zone, based on the “1” year as a time slice. **(D)** The top 25 keywords with the strongest citation bursts, with the red bold line representing the burst years.

The co-occurrence network, with 684 nodes and 969 links, reveals a mature intellectual structure (Figure 6A). Cluster analysis using the LLR algorithm identified 15 thematic groups (Q = 0.4939, S = 0.7714), confirming high modularity and cluster homogeneity. Key clusters include surgical techniques [e.g., orbital decompression (#5), rectus muscle transposition (#13), and mitomycin C application (#14)], pathophysiological mechanisms [genetic studies (#2) and disorders (#3)], and clinical evaluation [vision (#0), visual acuity (#10), and double blind (#7)] (Figure 6B).

The time-zone map (Figure 6C) illustrates the evolution of strabismus research themes, with a peak in thematic diversity during 2004–2006. Current research frontiers focus on AI-driven diagnostics, including deep learning for automated angle quantification and fusion function analysis, pediatric ophthalmic care addressing postoperative rehabilitation and preoperative anxiety, and the systemic disease link in Graves’ orbitopathy (GO), which significantly influences pathophysiology, diagnosis, and treatment. Burst analysis reveals sustained momentum in “refractive error” (strength: 25.24), “psychosocial aspects,” and “pediatric ophthalmology,” while emerging keywords such as “case report” and “Graves orbitopathy” (2021–2023) highlight a shift toward individualized management and systemic disease impacts (Figure 6D).

### Co-cited references and citation burst analysis

**Figure 7:**
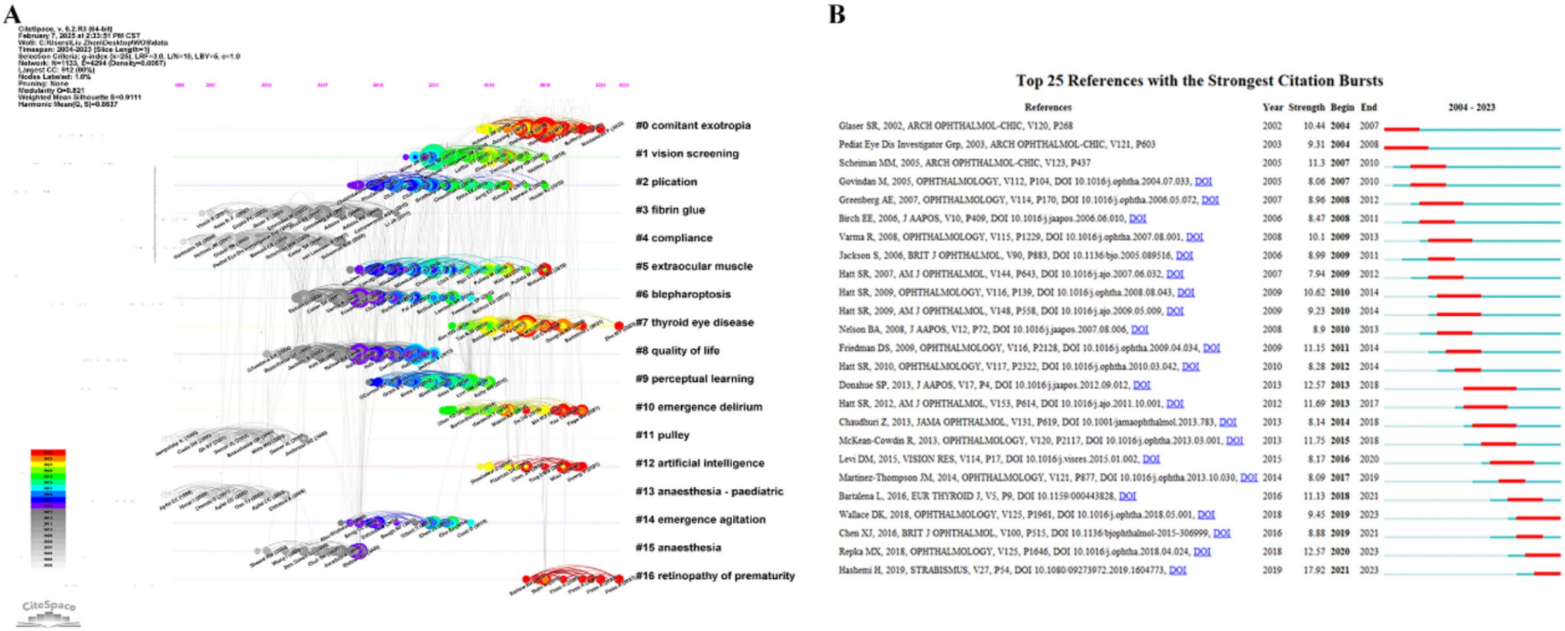
Reference co-citation analysis. **(A)** Timeline map of co-cited literature. The top of the image represents the timeline. Nodes represent references, and their distribution from left to right along the time axis indicates the year of the first publication of the cited literature, from earliest to most recent. # represents different cluster labels. **(B)** The top 25 references with the strongest citation bursts in the co-citation network.

The reference co-citation analysis provides insights into the evolution and interconnections within the scholarly literature on strabismus. Figure 7A presents a co-citation timeline, where the diameter of each node corresponds to the citation frequency, and the node’s color aligns with the chrono-spectral bar above. Warmer hues indicate more recent publications, while cooler hues represent earlier studies. Horizontal lines delineate different clusters, with adjacent labels describing the thematic focus of each cluster. This analysis identified highly cited studies, which are detailed in Table 5. Figure 7A shows a significant concentration of nodes within clusters #2, #3, #5, #6, #7, and #8, spanning an extensive temporal range. This distribution reflects sustained research interest in key areas such as “plication,” “fibrin glue,” “extraocular muscle,” “blepharoptosis,” “Graves orbitopathy,” and “quality of life” within the strabismus research community. These clusters underscore the enduring importance of these topics in both clinical and academic studies. In contrast, the emergence of clusters #0, #12, and #16, characterized by smaller and fewer nodes, suggests a growing interest in “comitant esotropia,” “artificial intelligence,” and “retinopathy of prematurity” in recent years. These emerging clusters highlight the shifting focus toward innovative and interdisciplinary research directions within the field.

**Table 5:** Top 10 references cited in the strabismus research field.

Rank	Frequency	References	DOI	Cluster ID
1	37	Hashemi H, 2019, STRABISMUS, V27, P54	10.1080/09273972.2019.1604773	0
2	35	Donahue SP, 2013, J AAPOS, V17, P4	10.1016/j.jaapos.2012.09.012	1
3	29	Repka MX, 2018, OPHTHALMOLOGY, V125, P1646	10.1016/j.ophtha.2018.04.024	7
4	28	Hatt SR, 2012, AM J OPHTHALMOL, V153, P614	10.1016/j.ajo.2011.10.001	8
5	25	McKean-Cowdin R, 2013, OPHTHALMOLOGY, V120, P2117	10.1016/j.ophtha.2013.03.001	1
6	25	Wallace DK, 2018, OPHTHALMOLOGY, V125, P1961	10.1016/j.ophtha.2018.05.001	0
7	24	Bartalena L, 2016, EUR THYROID J, V5, P9	10.1159/000443828	7
8	23	Hatt SR, 2009, OPHTHALMOLOGY, V116, P139	10.1016/j.ophtha.2008.08.043	8
9	22	Friedman DS, 2009, OPHTHALMOLOGY, V116, P2128	10.1016/j.ophtha.2009.04.034	6
10	21	Varma R, 2008, OPHTHALMOLOGY, V115, P1229	10.1016/j.ophtha.2007.08.001	6

The citation burst analysis of the 25 most influential references from 2002 to 2023 reveals critical inflection points in strabismus research (Figure 7B). The burst duration is indicated by a red line, while a dark blue line represents the citation timeline. Early foundational studies by Scheiman et al. (20) (strength = 11.3) established evidence-based frameworks for strabismus in children. A sustained increase in methodological rigor is evident from 2013 onward, as exemplified by Donahue et al. (21) (strength = 12.57) on the standardization of diagnostic criteria. These findings collectively illustrate the dynamic evolution of strabismus research, highlighting both longstanding areas of interest and emerging trends that are shaping the future of the field.

## Discussion

The bibliometric analysis of strabismus research has shown continuous growth since 2004, with the peak in annual publications reaching 341 in 2021. This growth trend reflects increasing attention to strabismus in both clinical and basic science domains, particularly in areas such as treatment innovations, the application of imaging technologies, and the impact of strabismus on visual development in children. Although there has been a decline in citation numbers, this phenomenon may be due to the limited time that newer publications have had to accumulate citations, particularly in emerging research areas such as the application of artificial intelligence in strabismus diagnosis. Overall, the sustained growth in strabismus research highlights the academic recognition and exploration of the field’s potential.

### Geographical distribution and institutional collaboration

The geographical distribution of strabismus research highlights the significant contributions of the USA, China, and the United Kingdom, with the USA leading in both research output and citation impact. This dominance reflects the robust research infrastructure, substantial funding, and collaborative networks within the USA, which have positioned it as a global hub for strabismus research. The UK, while producing fewer publications, demonstrates exceptional research quality, as evidenced by its high citation impact, highlighting its substantial academic influence in the field. Meanwhile, China has emerged as a key player, with institutions such as the Zhongshan Ophthalmic Center at Sun Yat-sen University showing a significant citation burst from 2020 to 2023, signaling its growing influence and research capabilities in this field. This result further validates the distinctive contributions and global impact of different countries/regions in strabismus research.

At the institutional level, US-based entities dominate the research landscape, with the University of California system, Harvard Medical School, and Mayo Clinic demonstrating particularly strong research activity. As one of the most prolific contributors to the field, the UC system’s work spans clinical innovation and technological integration, solidifying its leadership in academic research (22, 23). This diverse and interconnected collaborative network not only drives scientific progress but also provides valuable insights into future research directions and strategies.

### Research hotspots and emerging challenges

The analysis of keyword co-occurrence, citation bursts, and thematic clusters reveals a dynamic and evolving landscape in strabismus research, characterized by both established research hotspots and emerging challenges.

Surgical intervention remains a cornerstone of strabismus management, with ongoing advancements in techniques and materials. The prominence of keywords such as “strabismus surgery,” “rectus muscle transposition,” and “mitomycin C application” reflects a sustained interest in refining surgical outcomes. Orbital decompression (#5) and rectus muscle transposition (#13) are particularly notable for their role in addressing complex cases, such as those involving Thyroid eye disease (TED) and Graves orbitopathy (GO). The integration of novel materials such as fibrin glue and the application of mitomycin C to reduce postoperative fibrosis (#14) exemplifies efforts to enhance surgical precision and minimize complications. Pediatric strabismus continues to be a focal point, with keywords such as “children,” “pediatric ophthalmology,” and “postoperative visual rehabilitation” highlighting the unique challenges and opportunities in this population. The fragmented nature of research on pediatric populations, as indicated by the low centrality of “children,” suggests a need for more cohesive, large-scale studies to optimize diagnostic and therapeutic strategies. In addition, the intersection of strabismus and systemic diseases—particularly TED and GO—has emerged as a critical area of investigation. These conditions complicate strabismus management by altering extraocular muscle function and orbital dynamics. Addressing these challenges requires interdisciplinary collaboration between ophthalmologists, endocrinologists, and radiologists, marking an important shift toward integrated care in the management of strabismus in patients with systemic diseases.

A transformative shift is also underway in diagnostics, driven by the integration of AI and deep learning algorithms. Automated tools for strabismus angle quantification and fusion function analysis promise to enhance diagnostic accuracy, reduce inter-observer variability, and streamline clinical workflows. However, the adoption of AI faces several hurdles, including the need for robust, ethnically diverse datasets, validation in real-world settings, and resolution of ethical concerns surrounding data privacy and algorithmic bias. The increasing emphasis on “psychosocial aspects” and “quality of life” reflects a broader shift toward patient-centered care in strabismus research. Strabismus not only affects visual function but also has profound psychosocial implications, particularly in children and adolescents. Addressing these aspects requires a holistic approach that combines clinical interventions with psychological support and patient education.

In conclusion, the field of strabismus research is characterized by a rich interplay of established themes, such as surgical innovation, pediatric care, and systemic disease management, alongside emerging frontiers in AI-driven diagnostics and a heightened focus on psychosocial factors.

## Limitations

While this study provides a macroscopic view of strabismus research, several limitations warrant consideration. The reliance on the Web of Science database may underrepresent non-English publications, particularly from regions with emerging research output. Furthermore, bibliometric metrics inherently favor established authors and institutions, potentially overlooking groundbreaking contributions from smaller research groups. Future studies should integrate alternative data sources (e.g., clinical trial registries and patents) to capture translational innovations.

## Conclusion

In summary, the field of strabismus research has experienced significant development and growth over the past two decades. Future research on strabismus will place greater emphasis on interdisciplinary integration, spanning genetics to neurology, and clinical treatment to social impact. Research outcomes in these areas will further advance the diagnosis and treatment of strabismus. Precision medicine, personalized treatment plans, the application of new technologies, and interdisciplinary collaboration will be the core development directions for strabismus research and treatment. With ongoing technological advancements, early diagnosis, personalized treatment, and rehabilitation for strabismus will gradually become a reality, greatly improving patients’ quality of life.

## Data Availability

The raw data supporting the conclusions of this article will be made available by the authors without undue reservation.
